# Large-Scale Spraying of Roads with Water Contributes to, Rather Than Prevents, Air Pollution

**DOI:** 10.3390/toxics9060122

**Published:** 2021-05-28

**Authors:** Fengzhu Tan, Yuming Guo, Wei Zhang, Xingyan Xu, Ming Zhang, Fan Meng, Sicen Liu, Shanshan Li, Lidia Morawska

**Affiliations:** 1 Department of Environmental and Occupational Health, School of Public Health, Hebei Medical University, Shijiazhuang 050017, China; fjmuxxy@163.com (X.X.); zhangming2979@126.com (M.Z.); fanmeng818@163.com (F.M.); liusicenv@126.com (S.L.); 2 Climate, Air Quality Research Unit, Department of Epidemiology and Preventive Medicine, School of Public Health and Preventive Medicine, Monash University, Melbourne, VIC 3004, Australia; yuming.guo@monash.edu (Y.G.); shanshan.li@monash.edu (S.L.); 3 Chongqing Institute of Green and Intelligent Technology, Chinese Academy of Sciences, Chongqing 400714, China; andyzhangwei@163.com; 4 International Laboratory for Air Quality and Health, Queensland University of Technology, 2 George Street, Brisbane, QLD 4000, Australia; l.morawska@qut.edu.au

**Keywords:** fine particles, aerosols, air pollution, relative humidity, air pollution events, SNA

## Abstract

Spraying roads with water on a large scale in Chinese cities is one of the supplementary precaution or mitigation actions implemented to control severe air pollution events or heavy haze-fog events in which the mechanisms causing them are not yet fully understood. These air pollution events were usually characterized by higher air humidity. Therefore, there may be a link between this action and air pollution. In the present study, the impact of water spraying on the PM_2.5_ concentration and humidity in air was assessed by measuring chemical composition of the water, undertaking a simulated water spraying experiment, measuring residues and analyzing relevant data. We discovered that spraying large quantities of tap or river water on the roads leads to increased PM_2.5_ concentration and humidity, and that daily continuous spraying produces a cumulative effect on air pollution. Spraying the same amount of water produces greater increases in humidity and PM_2.5_ concentration during cool autumn and winter than during hot summer. Our results demonstrate that spraying roads with water increases, rather than decreases, the concentration of PM_2.5_ and thus is a new source of anthropogenic aerosol and air pollution. The higher vapor content and resultant humidity most likely create unfavorable meteorological conditions for the dispersion of air pollution in autumn and winter with low temperature.

## 1. Introduction

Numerous severe air pollution events have occurred in China since 2013. For example, in the Beijing, Tianjin and Hebei (BTH) region, severe air pollution events occurred during 21.7 days in January 2013 [1,2,3,4,5,6,7,8]. Even though there was little motor vehicle traffic and the suspended industrial production during the COVID-19 epidemic, some cities still encountered severe air pollution events (BTH occurred two large-scale air pollution events on January 23–28 and February 8–13) [9,10,11]. Many studies have shown that severe air pollution events have the following common characteristics: (1) These events occur frequently in autumn and winter [1,2,5,12,13,14,15,16]. (2) These events are accompanied by certain unfavorable meteorological conditions: low temperatures, calm winds, inversions, and high humidity (sometimes exceeding 90%) [1,6,10,11,14,17,18,19,20,21,22,23]. (3) They are recurring, and a typical severe air pollution event lasts approximately 6–9 days and can be divided into three stages: an accumulative rising stage (PM_2.5_ rising from 50 to 200 μg/m^3^ and humidity reaching 60%, lasting for 3–4 days); the peak stage (sharp increases in the PM_2.5_ level, sometimes increasing by several times in 1 h and reaching concentrations as high as 700 μg/m^3^; humidity exceeding 80%; lasting for 2–3 days); and a declining stage (featuring a cold wind or rain and decreases in both PM_2.5_ and humidity, returning to sunny conditions within 1 day). Each cycle is broken by wind or rain [4,8,17,19,21,22,24,25,26]. (4) These events often have a rapid onset and disappearance. Approximately 45% or more of the peak stage formed rapidly within 1–9 h, and approximately 50% of severe air pollution events quickly declined from the sixth pollution level (AQI > 300) to the second pollution level (AQI 51–100) within 1–8 h [4,14,19,21,25,27,28]. (5) The haze-fog itself is mainly composed of inorganic aerosols. Water-soluble sulfates, nitrates and ammonium (SNA) account for 35–60% of PM_2.5_ and are mostly in liquid-droplet form in the aqueous phase. The chemical composition of heavy haze-fog in the BTH region is basically the same [1,6,7,9,10,11,21,22,25,26,29,30,31,32,33,34].

The following primary factors are considered the cause of severe air pollution events: (1) Increased particulate emissions (mainly from industrial coal combustion and winter heating, and fuel consumption in transportation) [35,36,37]. (2) An unfavorable geographical location for air pollutant diffusion (specifically, the dustpan-like terrain in the BTH region) [24]. (3) The unfavorable meteorological conditions mentioned above and (4) interregional transfer (nearly 60% of particulate matter in Beijing is considered to come from the southwest direction of Hebei) [28,38,39].

In response to the frequent occurrence of severe air pollution events, various strict control measures have been implemented (energy conservation and emission reduction, clean energy development, shutdown of heavily polluting enterprises, motor vehicle number control, road driving restrictions, and the use of gas instead of coal etc.) [40,41]. Spraying water on roads to reduce dust or particulate matter is also one of these supplementary precautions. Hence, municipalities ranging in size from large cities to small county towns have been equipped with water-spraying and fog-spraying trucks (sprayers; Figure S1). However, the improvement in air quality has been unsatisfactory. The frequent occurrence of severe air pollution events in autumn and winter remains poorly understood. It is unclear why severe air pollution events are still so common after implementation of these measures. Aerial photographs of different cities (Figure S2) reveal that the grey-white haze-fog was close to ground level and did not exceed the height of residential buildings (generally 33 floors and 100 m high). One gradient monitoring study showed that PM_2.5_ concentrations were highest at 86 m above the ground [42]. This observation inspired us to ask the question: where did the grey-white haze-fog come from in cities full of many high-rise buildings and asphalt roads? Why did these events still occur under the situation of few motor vehicles traffic and the suspended industrial production during the COVID-19 epidemic? As fog is formed by the condensation of water vapor [43], we should first explore the development of conditions associated with condensation (condensation nuclei, high humidity, cold air, low temperature, etc.).

In this study, using water composition measurements, water-spraying simulation experiments and data analysis, we attempt to explore the following questions: Does spraying water on roads actually decrease air PM_2.5_ concentrations? Does it change the air humidity? Could spraying be a contributing factor to the formation of severe air pollution events? Our results will provide a basis for explaining the formation, prevention and control of severe air pollution events, and for researching the effects of anthropogenic aerosol on regional climates.

## 2. Materials and Methods

Experimental design: The purpose of all experiments was to explore and determine whether spraying water on roads increases air PM_2.5_ concentration and humidity. The water used to spray the roads is normally tap water or river water. Consequently, these two types of water were used in two separate experiments, while ultrapure water was used as a control. The experiments controlled the confounding factors that may affect PM_2.5_ concentration on roads (wind, humidity, temperature, air pressure, automobile exhaust and other air pollutants), and simulated the process of aerosol formation during water spraying, and the process of water evaporation and residue formation.

Calculation basis: In Y city, there are more than 100 water-spraying trucks or water sprayers (not including fog-spraying trucks or fog sprayers). The total amount of sprayed water per day is 8000 t (each water sprayer carries 20 t of water and sprays four times a day); 40,000 t of water is sprayed over the course of 5 days. The city’s main urban area is approximately 150 km^2^, and heavy haze-fog usually extends from the surface to a height of 100 m (Figure S2); therefore, the total volume of air used is calculated as follows: 

Y city’s main urban area (150 km^2^) × 100 m height = 1.5 × 10^10^ m^3^.

Statistical analysis: All statistical analyses were performed using SAS software (Contract Site Number: 553024).

### 2.1. Water Composition Measurement

The purpose of the water composition measurement was to identify the main chemicals in the water and to estimate the amount added to the road surface and air after spraying water on the roads. Sulfate, nitrate and chloride were measured with ion chromatography (Thermo Electron Corporation, Waltham, MA, USA); lead, cadmium, arsenic, sodium, nickel and manganese were measured with inductively coupled plasma mass spectrometry (ICP-MS, Perkin Elmer, Waltham, MA, USA); chromium, ammonia nitrogen, chromaticity, turbidity, total hardness, total dissolved solids and total number of colonies were measured by the relevant standard methods [44].

### 2.2. Simulated Water Spraying Experiment

#### 2.2.1. Five-Day Humidifying Experiment 

The purpose of the five-day humidifying experiment was to investigate whether continuous humidification increases the air PM_2.5_ concentration and humidity, and their duration, by simulating the process of road spraying water and atomization or aerosolization (assuming that the sprayed water is completely aerosolized). The amount of water required for one chamber and one experiment was calculated using the air temperature and saturated humidity. It was necessary to do pre-experiments in order to assess a cumulative effect of fine particles and humidity just like the cumulative characteristics in severe air pollution events. Before each experiment, the PM_2.5_ in each chamber was removed by an air purifier, and the humidity was reduced by a dehumidifier. Then, humidifiers containing river water, tap water and ultrapure water were placed in 3 different sealed chambers, respectively, which were humidified for 40 min a day for 5 days (250 mL of water in total). The PM_2.5_ concentration, air temperature and humidity in each sealed chamber and in the surrounding room were measured and recorded before humidification and at different time points. Each parameter was measured twice and the mean of the two values was taken as the final measurement value. The experiment was repeated four times (N = 4) for each water type. The physical phenomena in each chamber during the experiment were also observed and recorded. The PM_2.5_ concentration was automatically measured (a laser particulate meter, Beijing JDHS Technology Co. Ltd, LD-5, Beijing, China), and the temperature and humidity were automatically measured and read (a digital device, Vaisala, HM 34, Tokyo, Japan). Differences in the PM_2.5_ concentration and humidity between the groups were analyzed using a general linear model. In addition, the wind speed, air temperature, pressure and volume, and the time and speed of humidification in the three chambers were the same or similar in our experiments, so that the only factor affecting the PM_2.5_ concentration and humidity was the type or components of water.

#### 2.2.2. One-Time Humidifying Experiment

The purpose of the one-time humidifying experiment was to investigate the increase in PM_2.5_ concentration and humidity, and their duration, after one humidification event involving a large amount of water. The method was generally the same as the 5-day experiment but involved the release of all 250 mL of water over the course of 200 min.

### 2.3. Water Total Residue Measurement and Analysis

#### 2.3.1. Residue Measurement

The purpose of the residue measurement was to estimate the amount of solid residue added to road surface and air by simulating the process of residues remaining after water evaporation on the road (assuming that the sprayed water is all left on the road). We used the constant weight method [44], which consisted of placing a 50 mL water sample in an evaporating dish at 77–77.3 °C in a thermostatic water bath, oven-drying for 1 h at 105 °C, cooling for 30 min, and weighing to a constant weight on an electronic balance. This was performed once a day for 5 days (250 mL water in total). Each experiment set was repeated three times (*N* = 3). The total residue was calculated as follows:

Total residue (mg/L) = residue and evaporating dish weight (g) − evaporating dish net weight (g) × 1000 × 1000/water sample volume (mL). 

The difference in residue weight between groups was analyzed using a general linear model. The road residue and air PM_2.5_ after evaporation of the water were estimated based on the total amount of sprayed water, the residue weight and the total volume of air (1.5 × 10^10^ m^3^).

#### 2.3.2. Residue Composition Analysis

The purpose of the residue composition analysis was to explore whether components in the residue were consistent with the components in the water. The residue remaining after evaporation was observed and imaged with electron scanning (Scanning Electron Microscope, SEM, Hitachi S-4800, Tokyo, Japan), and micro-area chemical element analysis was performed with spectroscopy (Energy Dispersive Spectrometer, EDS, INCA Energy 350, Oxford, England).

### 2.4. Impact of Spraying Water on Air Humidity

The impact of spraying different quantities of water on the increase in relative humidity (RH) at different temperatures was analyzed. The water content per cubic meter of air and the relative humidity when different amounts of water were sprayed on the roads were calculated according to the saturated humidity at different air temperatures, and the total volume of air (1.5 × 10^10^ m^3^). The difference in humidity between the groups was analyzed using a general linear model.

### 2.5. Relationship of PM_2.5_ to Air Temperature and Humidity

Based on data from Y city, air temperature and humidity characteristics at different PM_2.5_ concentrations were analyzed to explore the temperature and humidity conditions under which severe air pollution events are most likely to occur. The difference between days with different PM_2.5_ concentration was analyzed with a chi-square test (trend χ^2^), and non-conditional logistic regression was used to calculate the odds ratio (OR) and 95% CI (confidence interval) to further evaluate the risk of meteorological factors increasing the days of above-moderate pollution (PM_2.5_ ≥ 150 μg/m³). The variables were assigned as follows: temperature ≥ 10 °C = 0, temperature < 10 °C = 1; humidity < 56% = 0, humidity ≥ 56% = 1; PM_2.5_ < 150 μg/m^3^ = 0, PM_2.5_ ≥ 150 μg/m^3^ = 1. Wind speed and air pressure used their original values.

## 3. Results

### 3.1. Water Composition Measurement

Table 1 provides the amounts of the measured chemical components in water and the estimated values of these components on the road surface and in the air after spraying. For example, the chemical composition of tap water in decreasing order of abundance was total dissolved solids, calcium carbonate, sulfate, chloride, sodium and nitrate, and the concentrations of manganese, nickel, lead, cadmium, chromium and arsenic were low. If 8000 t of water were sprayed daily, the road surface and the air accumulated 652 kg and 43.5 μg/m^3^ sulfate, 233.6 kg and 15.6 μg/m^3^ chloride, and 41.92 kg and 2.8 μg/m^3^ nitrate, respectively. Compared with tap water, the river water contained higher concentrations of bacteria, ammonia nitrogen (which increased by 120 kg and 8.0 μg/m^3^ on the road and in the air, respectively) and manganese, but lower concentrations of nitrate (note that ammonia, nitrite and nitrate can change over time). The results suggest that both tap water and river water contain sulfate, ammonia, nitrates, and other compounds, and these components are expected to inevitably end up on the road surface or in the air when the two types of water are sprayed on roads. 

### 3.2. Simulated Water Spraying Experiment

#### 3.2.1. Five-Day Humidification Experiments

**Figure 1:**
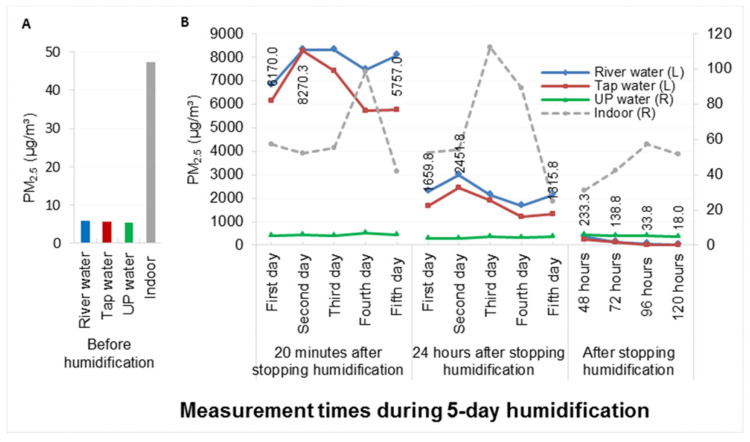
PM_2.5_ concentration in three sealed chambers and in the surrounding room before humidification (**A**) and after stopping humidification (**B**) in a five-day humidifying experiment. In panel B, PM_2.5_ concentrations between three types of water were different (DF = 2, F = 61.06, *p* < 0.0001), and PM_2.5_ concentrations between different time points (excluding ultrapure water) were also different (DF = 13, F = 55.5, *p* < 0.0001). Further multiple comparisons (LSD) showed that PM_2.5_ concentrations in tap water and river water were higher than those in ultrapure water (*p* < 0.05), but there was no difference in PM_2.5_ concentrations between tap water and river water (*p* > 0.05); the order of average PM_2.5_ concentrations between different time points was 20 min > 24 h > 48–120 h (*p* < 0.05). PM_2.5_ concentration decreased to a minimum on the fifth day after humidification ended. The marked numbers are the tap water results.

Figure 1 shows the PM_2.5_ concentration at different time points. For tap water, the PM_2.5_ concentration averaged only 5.5 μg/m^3^ before humidification (A). After the humidifying process ceased (B), the PM_2.5_ concentration increased from the first day to the second day (i.e., ranging from 6170.0 to 8270.3 μg/m^3^ at 20 min and from 1659.8 to 2451.8 μg/m^3^ at 24 h). However, the PM_2.5_ concentration started to decrease on the third day (possibly due to droplet formation on the inside wall of the sealed chamber) and was 1315.8 μg/m^3^ on the fifth day at 24 h. The PM_2.5_ concentration remained at 233.3 μg/m^3^ at 48 h and averaged 90.25 μg/m^3^ from 48 to 120 h (110.5 μg/m^3^ for river water). In panel B, the PM_2.5_ concentrations for both river water and tap water were higher than those for ultrapure water at each time point (see legend). The PM_2.5_ concentration in each sealed chamber was not affected by the PM_2.5_ concentration in the surrounding room. These results showed that continuous humidification with tap water or river water increased the PM_2.5_ concentration in the air and that the effect was cumulative. In addition, RH, temperature and physical changes at different time points were also recorded (see Figure S3).

#### 3.2.2. One-Time Humidifying Experiment

The results of this experiment are shown in Figures S4 and S5.

In addition, the field measurements were performed twice, but it was difficult to precisely measure the PM_2.5_ concentrations, humidity and temperature immediately after water was sprayed on the road because of the rapid evaporation of the water and the considerable influence of wind, vehicles, sunshine and automobile exhaust, etc.

### 3.3. Water Total Residue Measurement and Analysis

#### 3.3.1. Residue Measurement

**Figure 2:**
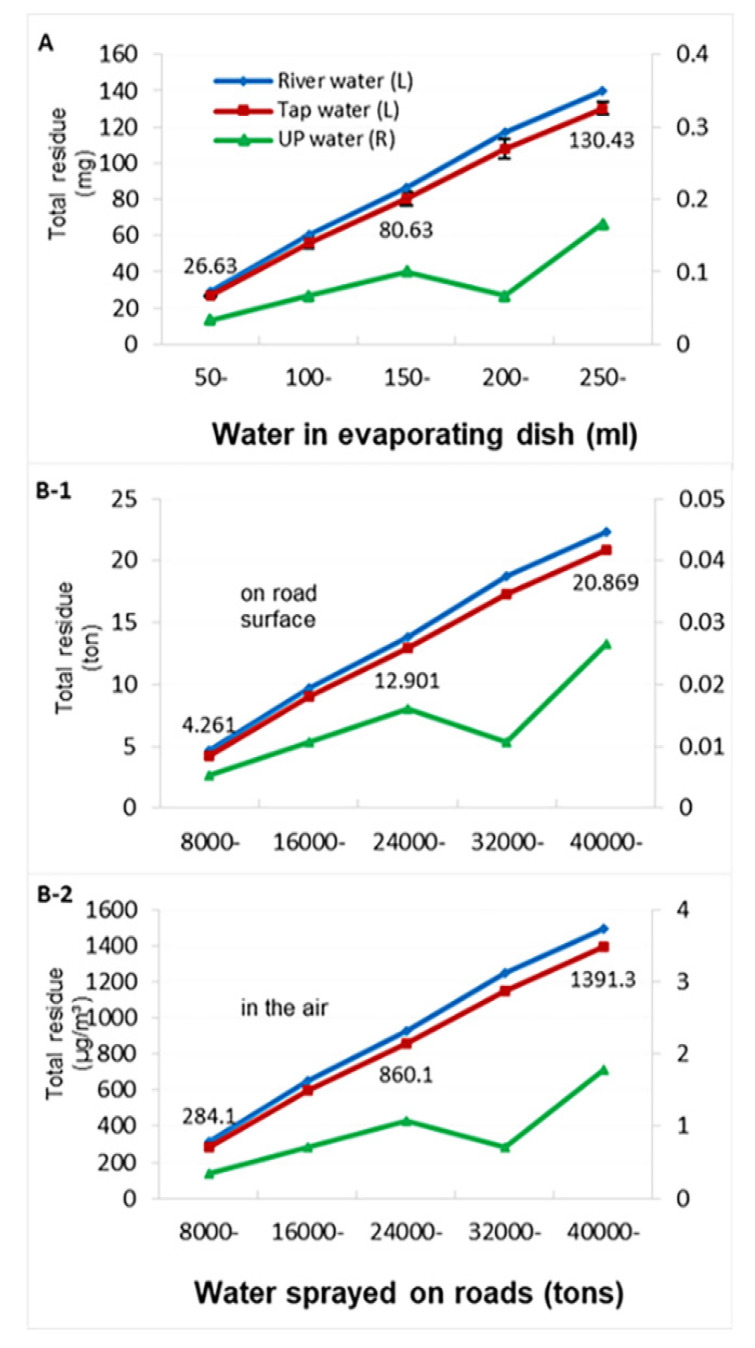
Total solid residue in the evaporating dish (**A**) for the three types of water and the estimated values of the residue on the road surface (**B-1**) and in the air (**B-2**). **A**: Evaporating 50 mL of water once a day for 5 d; each value on the curve was the mean from three experiments for each water sample. **B-1** and **B-2**: Calculated from 8000 tons of water sprayed on roads during one day and the total volume of air (1.5 × 10^10^ m^3^). In panel **A**, the means of the residue between the three types of water were different (DF = 2, F = 90.79, *p* < 0.0001), and the means of the residue between the five quantities of water were also different (DF = 4, F = 18.75, *p* < 0.0001). Further multiple comparisons (LSD) showed that the residues in tap water and river water were higher than those in ultrapure water (*p* < 0.05), but there was no difference in the residues between tap water and river water (*p* > 0.05); the order of the average residue values between different water quantities was 200 and 250 mL >100 and 150 mL > 50 mL (*p* < 0.05). The marked numbers are the tap water results.

Figure 2 shows the total solid residue in the evaporating dish (A) after water evaporation and the estimated values on the road surface (B-1) and in the air (B-2). For tap water, the total residue from 50 mL of water on the first day was 26.63 mg, and the total residue from 250 mL on the fifth day had reached 130.43 mg. Accordingly, spraying 8000 t of water on roads on the first day would add 4.261 t of total residue to the road surface and 284.1 μg/m^3^ of residue particles to the air (similar to the results in Table 1). Thus, spraying 40,000 t of water over five days would contribute 20.869 t of total residue on the road surface and 1391.3 μg/m^3^ to the air. The mean amounts of residue differed between the three types of water and the five levels of water quantity; the residue in tap water and river water were higher than those in ultrapure water (see legend). The road surface residue produced by spraying 8000 t of water per day is equivalent to the weight of dust emitted by burning 782 t of coal per day (calculated based on 6 kg of dust produced by an industrial boiler burning 1 t of coal). The above results show that the residues are likely to remain and accumulate on the road surface after the water sprayed on roads evaporates. In addition, it was found that the tap water residue easily absorbed moisture and adhered together, and that the river water residue was a light earthy yellow in color.

#### 3.3.2. Residue Composition Analysis

The result of this experiment can be found in Figure S6.

### 3.4. Impact of Spraying Water on Air Humidity

**Figure 3:**
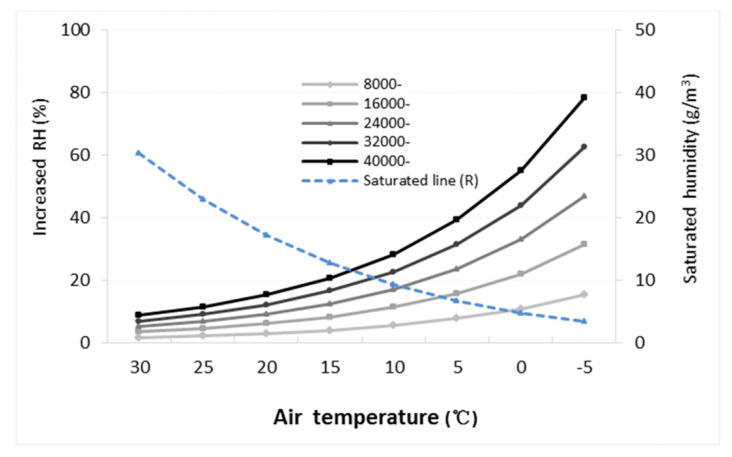
The increase in the relative humidity (RH) in the air after spraying water on roads for 5 days at different air temperatures. The RH varied between different quantities of water sprayed on the roads (DF = 4, F = 14.21, *p* < 0.0001), and between different air temperatures (DF = 7, F = 17.95, *p* < 0.0001). Further multiple comparisons (LSD) showed that the RH on the fourth and fifth day (i.e., 32,000 and 40,000 t of water) was higher than that on the first and second day (i.e., 8000 and 16,000 t of water, *p* < 0.05), but there was no difference between 8000 and 24,000 t of water. The RH at air temperatures of 5, 0 and –5 °C was higher than that at 25 and 30 °C (*p* < 0.05).

Figure 3 shows the air RH changes with different water quantities at different air temperatures. The air RH gradually increased as the air temperature decreased and the amount of sprayed water increased. When the air temperature was 30 °C, spraying 8000 t of water on the first day increased the RH by only 1.75%, and spraying a cumulative 40,000 t of water over the course of five days increased the RH by only 8.81%. However, when the air temperature was 0 °C, spraying 8000 t of water increased the RH by 10.93%, and cumulatively spraying 40,000 t of water would increase the RH by 55.05%. In particular, the increased RH was different at different air temperatures and sprayed water quantities (see legend). Undoubtedly, this elevated RH (i.e., water vapor content), together with low temperatures in autumn and winter, would result in meteorological conditions unfavorable for air pollutant diffusion.

### 3.5. Relationship of PM_2.5_ to Air Temperature and Humidity

Table 2 shows the relational analysis between PM_2.5_ concentration and air temperature and humidity. Days with higher PM_2.5_ pollution levels were more common and gradually increased at temperatures < 10 °C and RH ≥ 56% (*p <* 0.0001). Days with heavy haze-fog corresponding to the highest PM_2.5_ pollution level (i.e., the 300 μg/m^3^ group, with an average concentration of 426.3 μg/m^3^) accounted for 39.2% of its group (31/79) and 83.8% of the year (31/37). In contrast, days with heavy haze-fog at temperatures < 10 °C and RH values < 56% accounted for only 3.6% (2/56) of the group and 5.4% (2/37) over the year. 

Further analysis on the group of temperatures < 10 °C showed that severe air pollution events mainly occurred at temperatures < 5.0 °C and RH ≥ 56% (days with heavy haze-fog still were 31 days), which is consistent with the rapid increase and exacerbation of RH when water was sprayed on roads at air temperatures of 5 to −5 °C (Figure 3). These results indicate that severe air pollution events commonly occurred in autumn and winter because of the low temperatures and high humidity. In addition, the total number of days in the 200 and 250 μg/m^3^ groups accounted for 43.4% of the year [(9+14) / (26+27)]. However, in the remaining temperature and RH groups, the number of days in the different PM_2.5_ groups gradually decreased (*p* < 0.0001).

Multivariate logistic regression showed that the OR (95% CI) of increase in the days of above-moderate pollution (PM_2.5_ ≥ 150 μg/m³) for high humidity, low temperature, high air pressure and low wind speed were 9.957 (4.770–20.785), 2.890 (1.360–6.142), 1.006 (1.002–1.010) and 0.942 (0.896–0.990); their corresponding attributable risk percentages (AR%) were 89.96%, 65.40%, 0.60% and -6.16%, respectively. These results indicate that high humidity (≥ 56%), low temperature (< 10 °C) and high air pressure are favorable conditions for the occurrence of severe air pollution events, but high wind speed is not.

## 4. Discussion

Most studies of Chinese severe air pollution events come from China. These studies have shown that the frequent heavy haze-fog is characterized by high concentrations of water-soluble SNA particles [6,13,14,20,21,22,33], and have concluded that unfavorable meteorological and geographical conditions are the main causes of severe air pollution events [1,6,14,17,18,19,20,21,22,23,24]. One climate model simulation [45] indicated that boreal cryospheric forcing enhanced the regional circulation mode of poor ventilation in the East China plains region and provided conditions conducive to the formation of extreme haze events, such as those in 2013. However, if this hypothesis is correct, why did the extreme haze not occur in other countries?

There is no simulated water spraying experiment like ours so far although there are two smog chamber studies related to vehicle exhaust [46] and residential coal combustion [47]. Few studies have considered why these regional meteorological conditions have become so severe. Why is the air humidity so high? Where did the large amount of water vapor come from? Frequent severe air pollution events in urban areas no longer feature a direct causal link with industrial production after strict pollution control measures were implemented, so why do severe air pollution events still occur frequently? For example, four severe air pollution events occurred in November and early December of 2018 in China and were not limited to the BTH region; especially, these events still occurred in some regions under the situation of few motor vehicles traffic and the suspended industrial production during the COVID-19 epidemic [9,10,11]; consequently, there must be a common special cause across the country.

Spraying water into the air was initially applied to remove dust at mining and construction sites. Now, spraying water on roads has been used as a precaution to reduce dust and haze in various cities and counties in China [41,48]. However, our results suggest that this measure does not actually reduce the concentration of PM_2.5_ in the air for the following reasons.

Unlike ultrapure water, tap water (often purified in waterworks) and river water (often with more bacteria) contain more minerals and soluble salts, among other components (as detailed in Table 1 and Figure S6). These components are mostly consistent with some of the inorganic particles in heavy haze-fog occurring in autumn and winter [1,6,22,25,26,29,30,31,32,33,34].

Spraying water on a road with a high-pressure nozzle causes atomization of any substances suspended or dissolved in the water. The spraying process results in aerosolization of large numbers of water droplets and produces water mist (Figure S1), which is similar to the situation in which sea salt aerosols are generated from seawater spindrift [49,50]. Many substances dissolved or suspended in the water can be dispersed directly into the air along with the droplets. These aerosol particles range in size from 1 μm to tens of μm, with the actual size distribution depending strongly on many factors but particularly on the velocity of air passing through the aerosolized liquid. The size distribution of the initially formed droplets can be calculated if the concentrations of the substances and the air velocity are known, but this would be difficult with complex mixtures of components. After aerosolization, the fate of droplets depends on their size and the RH (the lifespan of water vapor and aerosol is about 10 days, and that of ammonium sulfate is approximately 6 days) [43,49]. All the droplets will start losing some water content by evaporation (unless RH is 100%). The larger droplets will immediately be deposited by gravitation. The smaller droplets, however, will stay suspended in the air, becoming even smaller with evaporation, adding to the existing PM_2.5_ levels. In addition, the water on the road evaporates quickly within 15–30 min, and the minerals and salts dissolved in the water may remain on the road surface and form fine particles (Figure 2 and Figure S6), which is similar to the way sea salt is produced after seawater exposed to the sun evaporates in beach salt ponds. These dry and invisible fine particles could be resuspended in the air by the instantaneous turbulence caused by vehicles even when there is no wind in the atmosphere. This process is similar to the mechanism by which dust is raised by vehicles crossing dirt road in the absence of wind. This is the addition to PM_2.5_ remaining after the sprayed water on the road evaporates.

The increase in RH is related to the increase in water vapor in the air and the decrease in air temperature. In the process of spraying water on roads, water is first introduced into the air by aerosolization (droplets), which thus increases the air humidity. Secondly, the evaporation of water on the road surface leads to an increase in water vapor, which further increases the air humidity. The maximum amount of water vapor (the saturated humidity) accommodated by 1 m^3^ of dry air varies at different temperatures (a physical principle) [49,51]. If the amount of water sprayed in the cold autumn and winter is the same as that in hot summer conditions, the RH will rapidly increase to a greater degree in the cold conditions than in the hot conditions (Figure 3). This is the addition to RH as a result of spraying and water evaporation.

It could be said that spraying water on the roads to reduce PM_2.5_ concentration actually results in the opposite effect to that intended, considering the scale of spraying water, and also involves significant costs.

## 5. Conclusions

The present study explored the impact of spraying roads with water on PM_2.5_ concentrations and humidity in the air. The spraying process, water evaporation, and the remaining residues all contribute to an additional increase in anthropogenic aerosol or PM_2.5_ and humidity. The same amount of water sprayed on days with low temperatures and calm wind, especially during daily continuous water spraying, may produce a greater increase in PM_2.5_ concentration and humidity in the cold autumn and winter than in the hot summer. Daily spraying of water on roads does not reduce PM_2.5_ concentrations in the air. Instead, the sprayed water may produce new anthropogenic aerosol or invisible fine particles and thus become a new source of air pollution. Undoubtedly, the increased anthropogenic aerosols, together with low temperatures in autumn and winter, will promote the formation of high-humidity meteorological conditions unfavorable for the air pollutant diffusion, and become the main cause of severe air pollution events in low-temperature weather.

## Figures and Tables

**Table 1:** The measured amounts for chemical components in the three types of water and their estimated values on the road surface and in the air.

Measurement Parameters	Measurement Results (mg/L) ^†^			Amount Added to Roads (kg) ^‡^			Amount Added to the Air (μg/m³) ^#^		
	River Water	Tap Water	UP Water	River Water	Tap Water	UP Water	River Water	Tap Water	UP Water
Total number of colonies (CFU/mL)	2700	no	no	-	-	-	-	-	-
Chromaticity (Platinum cobalt color unit)	Light earth yellow	<5	<5	-	-	-	-	-	-
Turbidity (NTU)	20	<1	<1	-	-	-	-	-	-
Total dissolved solids	541	407	21	4328	3256	168	288.5	217.1	11.2
Total hardness (calculated as CaCO_3_)	321	250	5	2568	2000	40	171.2	133.3	2.7
Sulfate	123	81.5	0.23	984	652	1.84	65.6	43.5	0.1
Chloride	86	29.2	0.74	688	233.6	5.92	45.9	15.6	0.4
Ammonia nitrogen	15	<0.02	<0.02	120	<0.16	<0.16	8.0	0.0	0.0
Nitrate (calculated as nitrogen)	0.65	5.24	0.31	5.2	41.92	2.48	0.3	2.8	0.2
Sodium	53	16	0.9	424	128	7.2	28.3	8.5	0.5
Manganese	0.34	<0.01	<0.01	2.72	<0.08	<0.08	0.2	0.0	0.0
Nickel	0.009	0.0032	0.0002	0.072	0.0256	0.0018	0.0	0.0	0.0
Lead	<0.01	<0.01	<0.01	<0.08	<0.08	<0.08	0.0	0.0	0.0
Cadmium	<0.003	<0.003	<0.003	<0.024	<0.024	<0.024	0.0	0.0	0.0
Chromium	<0.004	<0.004	<0.004	<0.032	<0.032	<0.032	0.0	0.0	0.0
Arsenic	<0.003	<0.003	<0.003	<0.024	<0.024	<0.024	0.0	0.0	0.0

† The units are mg/L except for the total number of colonies, chromaticity and turbidity. ‡ Equal to the measured value multiplied by 8000 tons of water sprinkled on roads during one day (8000 tons = 100 (sprinklers) × 20 (load per sprinkler) × 4 (four times a day)). # Equal to the amount added to roads divided by total volume of air (1.5 × 10^10^ m^3^).

**Table 2:** PM_2.5_ relation to air temperature and relative humidity in y city in 2013.

TEMP (°C) ^†^	RH (%) ^†^	PM_2.5_ Groups (μg/m^3^)							
		<100--	100--	150--	200--	250--	300--	Total	*p* for Trend ^#^
TEMP < 10	≥56	2 *	11	12	9	14	31	79	<0.0001
		62.5 **	126.6	178.8	223.7	275.5	426.3	288.0	
	<56	32	12	5	2	3	2	56	
		60.8	121.6	176.2	234.0	265.3	421.0	114.1	
10 ≤ TEMP < 20	≥56	11	9	8	6	9	4	47	<0.0001
		69.5	125.0	167.4	221.7	269.8	345.8	178.1	
	<56	26	11	3	1	1	0	42	
		62.2	126.4	166.0	207.0	273.0		94.9	
TEMP ≥ 20	≥56	42	32	17	8	0	0	99	<0.0001
		66.4	125.7	174.5	227.4			117.2	
	<56	31	11	0	0	0	0	42	
		57.2	126.7					75.4	
Total		144	86	45	26	27	37	365	
		62.6	125.4	174.0	224.5	272.4	417.3	154.1	
TEMP < 5 ^‡^	≥56	1	7	8	7	13	31	67	<0.0001
		39.0	132.1	181.0	219.4	276.8	426.3	309.9	
	<56	17	2	2	1	2	2	26	
		61.2	131.5	183.5	243.0	265.0	421.0	126.4	
5 ≤ TEMP < 10 ^‡^	≥56	1	4	4	2	1	0	12	0.0059
		86.0	117.0	174.3	238.5	259.0		165.6	
	<56	15	10	3	1	1	0	30	
		60.3	119.6	171.3	225.0	266.0		103.5	
Total ^‡^		34	23	17	11	17	33	135	
		60.9	124.0	178.0	225.5	273.7	426.0	215.8	

^†^ TEMP: air temperature; RH: relative humidity. ^‡^ Further analysis for the air temperature <10°C group. * Days with different PM_2.5_ concentration groups. ** PM_2.5_ concentration (mean, μg/m^3^) for different days. ^#^ Trend test for different days with different relative humidity values but the same air temperature.

## Data Availability

Data is contained within the article.

## Supplementary Materials

The following are available online at https://www.mdpi.com/article/10.3390/toxics9060122/s1, Figure S1: Various dust-removal trucks in operation on roads. A. Water-spraying truck with a high-pressure nozzle. B. Wet road surface after spraying water in the morning. C. Fog-spraying truck with an atomizing device. D. Road sweeper with a high-pressure nozzle, Figure S2: Aerial photographs of grey-white haze-fog rising from the ground in different cities. In these urban areas with many high-rise buildings and asphalt roads and with few industrial enterprises emitting pollutants except for traffic pollution, where did the heavy haze-fog come from? Figure S3: Changes in RH (A1, A2) and temperature (B1, B2) and physical phenomena in each sealed chamber during the five-day humidifying experiments, Figure S4: PM_2.5_ concentration in three sealed chambers and in the surrounding room before humidification (A) and after stopping humidification (B) in one-time humidifying experiment, Figure S5: Changes in RH (C1, C2) and temperature (D1, D2) and physical phenomena in each sealed chamber in one-time humidifying experiment, Figure S6: Residue composition analysis in the three types of water. River water (A-1, A-2, A-3) contained 16 elements, and the atomic percentages were O (65.87%), C (22.77%), Si (4.88%), Ca (0.71%), and Al, Fe, Mg, Na, Cl, K, S, F, P, Zr, Zn, Ti (0.03%); tap water (B-1, B-2, B-3) mainly contained eight elements, and the atomic percentages were O (66.79%), C (21.69%), Ca (5.05%), S (4.88%), and Si, Al, Cl, Zr (0.07%).

## References

[1] Sun Z., Ma T., Zhu L., Duan F., He K. (2017). Characteristics and formation of heavy winter haze pollution during 2014–2015 in Tianjin, China. EGU General Assembly.

[2] Meng X., Yu Y., Zhang Z., Li G., Wang S., Du L. (2014). Preliminary study of the dense fog and haze events formation over Beijing-Tianjin-Hebei Region in Janunary of 2013. Environ. Sci Technol..

[3] Hu J., Duan F., He K., Ma Y., Dong S., Liu X. (2016). Characteristics and mixing state of S-rich particles in haze episodes in Beijing. Front. Environ. Sci. Eng..

[4] Gao J., Peng X., Chen G., Xu J., Shi G., Zhang Y., Feng Y. (2016). Insights into the chemical characterization and sources of PM_2.5_ in Beijing at a 1-h time resolution. Sci. Total Environ..

[5] Hou S., Tong S., Ge M., An J. (2016). Comparison of atmospheric nitrous acid during severe haze and clean periods in Beijing, China. Atmos. Environ..

[6] Liu Y., Wu Z., Wang Y., Xiao Y., Gu F., Zheng J., Tan T., Shang D., Wu Y., Zeng L. (2017). Submicrometer particles are in the liquid state during heavy haze episodes in the urban atmosphere of Beijing, China. Environ. Technol. Lett. Sci..

[7] Wang X., Zhou Y., Cheng S., Wang G. (2016). Characterization and regional transmission impact of water-soluble ions in PM_2.5_ during winter in typical cities. China Environ. Sci..

[8] Wei P., Ren Z., Wang W., Su F., Gao Q., Cheng S., Zhang Y. (2015). Analysis of meteorological conditions and formation mechanisms of lasting heavy air pollution in eastern China in October 2014. Res. Environ. Sci..

[9] Wang P., Chen K., Zhu S., Wang P., Zhang H. (2020). Severe air pollution events not avoided by reduced anthropogenic activities during COVID-19 outbreak. Resour. Conserv. Recy..

[10] Le T., Wang Y., Liu L., Yang J., Yung Y., Li G., Seinfeld J. (2020). Unexpected air pollution with marked emission reductions during the COVID-19 outbreak in China. Science.

[11] Zhao N., Wang G., Li G., Lang J., Zhang H. (2020). Air pollution episodes during the COVID-19 outbreak in the Beijing–Tianjin–Hebei region of China: An insight into the transport pathways and source distribution. Environ. Pollut..

[12] Wang J., Zhou M., Liu B., Wu J., Peng X., Zhang Y., Han S., Feng Y., Zhu T. (2016). Characterizationand source apportionment of size-segregated atmospheric particulate matter collected at ground level and from the urban canopy in Tianjin. Environ. Pollut..

[13] Wang J., Zhang J., Liu Z., Wu J., Zhang Y., Han S., Zheng X., Zhou L., Feng Y., Zhu T. (2017). Characterization of chemical compositions in size-segregated atmospheric particles during severe haze episodes in three mega-cities of China. Atmos. Res..

[14] Han S., Wu J., Zhang Y., Cai Z., Feng Y., Yao Q., Li X., Liu Y., Zhang M. (2014). Characteristics and formation mechanism of a winter haze-fog episode in Tianjin, China. Atmos. Environ..

[15] Zhang H., Hu J., Qi Y., Li C., Chen J., Wang X., He J., Wang S., Hao J., Zhang L. (2017). Emission characterization, environmental impact, and control measure of PM_2.5_ emitted from agricultural crop residue burning in China. J. Clean. Prod..

[16] Zhao M., Wang S., Tan J., Hua Y., Wu D., Hao J. (2016). Variation of urban atmospheric ammonia pollution and its relation with PM_2.5_ chemical property in winter of Beijing, China. Aerosol Air Qual. Res..

[17] Wang Y., Zhang Q., Jiang J., Zhou W., Wang B., He K., Duan F., Zhang Q., Philip S., Xie Y. (2013). Enhanced sulfate formation during China’s severe winter haze episode in January 2013 missing from current models. J. Geophys. Res. Atmos..

[18] Ye X., Song Y., Cai X., Zhang H. (2016). Study on the synoptic flow patterns and boundary layer process of the severe haze events over the North China Plain in January 2013. Atmos. Environ..

[19] Quan J., Tie X., Zhang Q., Liu Q., Li X., Gao Y., Zhao D. (2014). Characteristics of heavy aerosol pollution during the 2012–2013 winter in Beijing, China. Atmos. Environ..

[20] Gao J., Tian H., Cheng K., Lu L., Zheng M., Wang S., Hao J., Wang K., Hua S., Zhu C. (2015). The variation of chemical characteristics of PM_2.5_ and PM_10_ and formation causes during two haze pollution events in urban Beijing, China. Atmos. Environ..

[21] Chen Y., Tang L., Wang Z., Qin W., Ge S., Zhou H., Wei J., Zhang Y., Jiang R. (2015). Weather process and particulate pollution characteristics during a winter haze episode in Nanjing. Environ. Sci. Technol..

[22] Han B., Zhang R., Yang W., Bai Z., Ma Z., Zhang W. (2016). Heavy haze episodes in Beijing during January 2013: Inorganic ion chemistry and source analysis using highly time-resolved measurements rom an urban site. Sci. Total Environ..

[23] Liu J., Liu Z., Wen T., Guo J., Huang X., Qiao B., Wang L., Yang Y., Xu Z., Wang Y. (2016). Characteristics of size distribution of water soluble inorganic ions during a typical haze pollution in the autumn in Shijiazhuang. China Environ. Sci..

[24] Yang X., Zhou Y., Cheng S., Wang G., Wang X. (2016). Characteristics and formation mechanism of a heavy winter air pollution event in Beijing. China Environ. Sci..

[25] Li H., Duan F., He K., Ma Y., Kimoto T., Huang T. (2016). Size-dependent characterization of atmospheric particles during winter in Beijing. Atmosphere.

[26] Wang H., Li X., Shi G., Cao J., Li C., Yang F., Ma Y., He K. (2015). PM_2.5_ chemical compositions and aerosol optical properties in Beijing during the Late Fall. Atmosphere.

[27] Lin J., An J., Qu Y., Chen Y., Li Y., Tang Y., Wang F., Xiang W. (2016). Local and distant source contributions to secondary organic aerosol in the Beijing urban area in summer. Atmos. Environ..

[28] Yuan Q., Li W., Zhou S., Yang L., Chi J., Sui X., Wang W. (2015). Integrated evaluation of aerosols during haze-fog episodes at one regional background site in North China Plain. Atmos. Res..

[29] Sun Z., Mu Y., Liu Y., Shao L. (2013). A comparison study on airborne particles during haze days and non-haze days in Beijing. Sci. Total Environ..

[30] Huang X., Liu Z., Zhang J., Wen T., Ji D., Wang Y. (2016). Seasonal variation and secondary formation of size-segregated aerosol water-soluble inorganic ions during pollution episodes in Beijing. Atmos. Res..

[31] Wang Q., Ma Y., Tan J., Zheng N., Duan J., Sun Y., He K., Zhang Y. (2015). Characteristics of size-fractionated atmospheric metals and water-soluble metals in two typical episodes in Beijing. Atmos. Environ..

[32] Meng C., Wang L., Zhang F., Wei Z., Ma S., Ma X., Yang J. (2016). Characteristics of concentrations and water-soluble inorganic ions in PM_2.5_ in Handan City, Hebei province, China. Atmos. Res..

[33] Zhang Y., Huang W., Cai T., Fang D., Wang Y., Song J., Hu M., Zhang Y. (2016). Concentrations and chemical compositions of fine particles (PM_2.5_) during haze and non-haze days in Beijing. Atmos. Res..

[34] Zhang J., Wang L., Wang Y., Wang Y. (2016). Submicron aerosols during the Beijing Asia Pacific Economic Cooperation conference in 2014. Atmos. Environ..

[35] Li K., Liao H., Mao Y.H., Ridley D.A. (2016). Source sector and region contributions to concentration and directradiative forcing of black carbon in China. Atmos. Environ..

[36] Ma Q., Cai S., Wang S., Zhao B., Martin R.V., Brauer M., Cohe A.N., Jiang J., Zhou W., Hao J. (2017). Impacts of Coal Burning on Ambient PM_2.5_ Pollution in China. Suppl. Atmos. Chem. Phys..

[37] Wu Y., Zhang S., Hao J., Liu H., Wu X., Hu J., Walsh M.P., Wallington T.J., Zhang K.M., Stevanovic S. (2017). On-road vehicle emissions and their control in China: A review and outlook. Sci. Total Environ..

[38] Zhang Y., Ding A., Mao H., Nie W., Zhou D., Liu L., Huang X., Fu C. (2016). Impact of synoptic weather patterns and inter-decadal climate variability on air quality in the North China Plain during 1980e2013. Atmos. Environ..

[39] Li J., Han Z. (2016). A modeling study of severe winter haze events in Beijing and its neighboring regions. Atmos. Res..

[40] State Council of the People’s Republic of China Air Pollution Prevention and Control Action Plan 2013. http://www.gov.cn/zwgk/2013-09/12/content_2486773.htm.

[41] Ministry of Environmental Protection, People’s Republic of China (2013). Detailed Rules for Implementation of Action Plan for Prevention and Control of Atmospheric Pollution in Beijing, Tianjin, Hebei and the Surrounding Area. http://www.mee.gov.cn/gkml/hbb/bwj/201309/t20130918_260414.htm.

[42] Han J., Chen J., Qian W., Yue Y., Gao Q. (2016). The research on relationship between meteorological condition and atmospheric particles in Shijiazhuang. Environ. Monit. China.

[43] He J., Guo P., Yin Y., Shen S. (2015). An Introduction to Atmospheric Science.

[44] Ministry of Health, People’s Republic of China, Standardization Administration of the People’s Republic of China (2007). GB/T5750-2006 Standards Examination Methods for Drinking Water(S).

[45] Zou Y., Wang Y., Zhang Y., Koo J.H. (2017). Arctic sea ice, Eurasia snow, and extreme winter haze in China. Sci. Adv..

[46] Chang T.Y., Nance B.I., Kelly N.A. (2014). Modeling Smog Chamber Measurements of Vehicle Exhaust Reactivities. J. Air Waste Manag. Assoc..

[47] Geng C., Wang K., Wang W., Chen J., Liu X., Liu H. (2012). Smog chamber study on the evolution of fume from residential coal combustion. J. Environ. Sci..

[48] Amato F., Querol X., Johansson C., Nagl C., Alastuey A. (2010). A review on the effectiveness of street sweeping, washing and dust suppressants as urban PM control methods. Sci. Total Environ..

[49] Sheng P., Mao J., Li J., Ge Z. (2017). Atmospheric Physics.

[50] Zakey A.S., Giorgi F., Bi X. (2008). Modeling of sea salt in a regional climate model: Fluxes and radiative. J. Geophys. Res..

[51] Lutgens F.K., Tarbuck E.J., Tasa D.G. (2016). The Atmosphere: An Introduction to Meteorology.

