# Supplementary material for: Large-Scale Spraying of Roads with Water Contributes to, Rather Than Prevents, Air Pollution
Source: Toxics. 2021 May 28;9(6):122. doi: 10.3390/toxics9060122 (PMC8229925; doi:10.3390/toxics9060122)
Supplement: Supplementary file 1 [file toxics-09-00122-s001.zip › toxics-1228314-supplementary.pdf]

Article

Supplementary Materials: Large-Scale Spraying of Roads with Water Contributes to, Rather Than Prevents, Air Pollution

Fengzhu Tan, Yuming Guo, Wei Zhang, Xingyan Xu, Ming Zhang, Fan Meng, Sicen Liu, Shanshan Li and Lidia Morawska

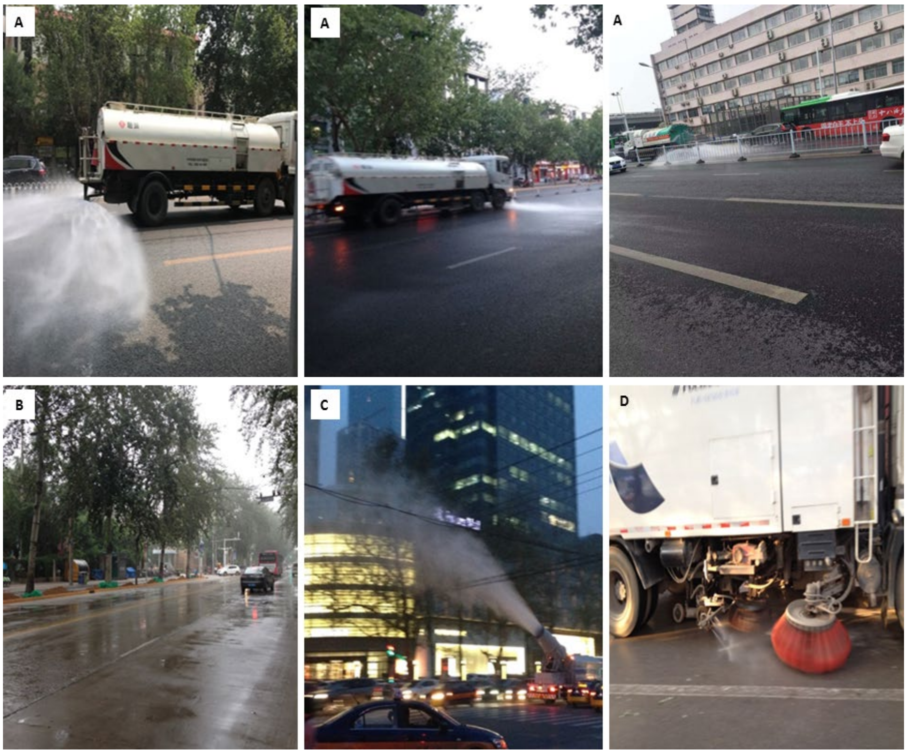

Figure 1. Various dust-removal trucks in operation on roads. **A**. Water-spraying truck with a high-pressure nozzle. **B**. Wet road surface after spraying water in the morning. **C**. Fog-spraying truck with an atomizing device. **D**. Road sweeper with a high-pressure nozzle.

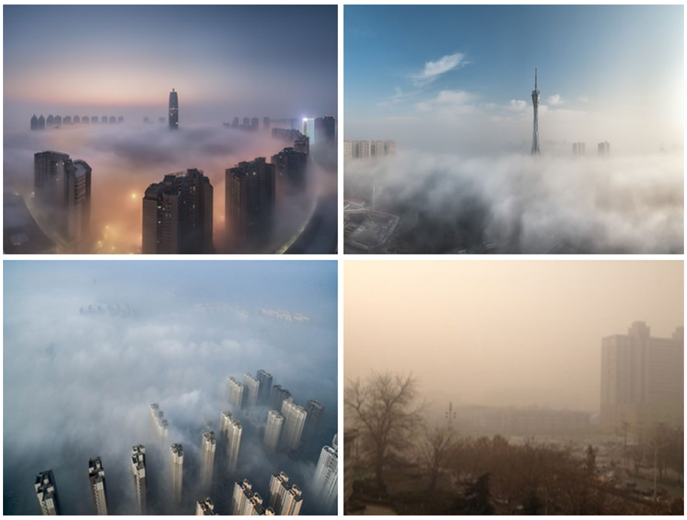

Figure 2. Aerial photographs of grey-white haze-fog rising from the ground in different cities. In these urban areas with many high-rise buildings and asphalt roads and with few industrial enterprises emitting pollutants except for traffic pollution, where did the heavy haze-fog come from?

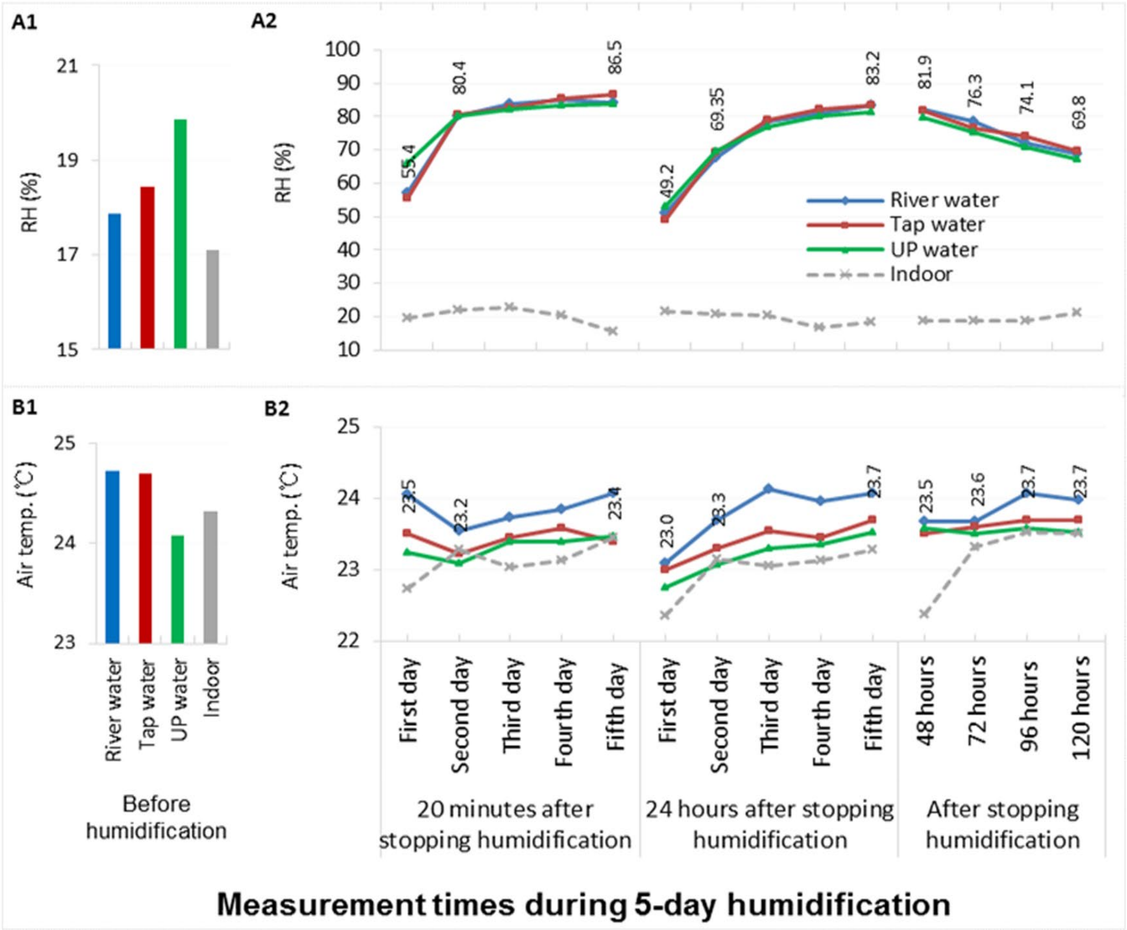

Figure 3. Changes in RH **(A1, A2)** and temperature **(B1, B2)** and physical phenomena in each sealed chamber during the five-day humidifying experiments.

For tap water, the RH averaged 20.3% before humidification **(A1)**. After stopping humidification **(A2)**, the RH increased from the first to the second day (i.e., from 55.4% to 80.4% at 20 minutes; from 49.2% to 69.35% at 24 hours), which was consistent with the increase in the $PM_{2.5}$ concentration in Figure 1. After that, the RH slowly increased on the second to fifth day. However, the RH decreased from 81.9% to 69.8% at 48-120 hours. The RH was no different between three types of water (DF=2, F=0.14, p=0.8762), but the RH at different measurement times was different (DF=13, F=29.63, p <0.0001). Further multiple comparisons (LSD) showed that the RH at 20 minutes on the fifth, fourth and third days, the RH at 24 hours on the fifth and fourth days and the RH at 48 hours were the highest, but the RH at 20 minutes and 24 hours on the first day was the lowest. The temperature (**B2**) was no different between three types of water and between different measurement times (DF=2, F=0.300, p=0.7396; DF=13, F=0.08, p=1, respectively). In addition, the RH and temperatures in the sealed chambers were not affected by those in the room. These results showed that humidification increased the air humidity and had a cumulative effect.

Physical phenomenon: Before humidification, each sealed chamber was dry, transparent, and lightly concave on side walls. At 20 minutes after stopping humidification, the sealed chambers showed no obvious changes on the first day; some fine droplets on the inside wall on the second day; light and medium fog, tiny droplets and slight expansion on the third day (this may be the reason for the $PM_{2.5}$ decrease); dense fog (which was denser for the river and tap water than for the ultrapure water), large droplets and moderate expansion on the 4th-5th days. At 24 hours, the fog disappeared, and the droplets decreased. The chambers exhibited moderate expansion. At 48-72 hours, the droplets further decreased, and the chambers moderately expanded. At 96-120 hours, the

droplets disappeared; chambers expanded by approximately 1/3 (an unexpected result) and were humid and hot. At end, there was an unpleasant odor in the river chamber.

The tap water and river water contain more minerals, soluble salts (sulfates and nitrates), and other components (as shown in Table 1), and these chemical components and fine water droplets will form $PM_{2.5}$ or aerosols when the water was sprayed into the air. The disappearance of $PM_{2.5}$ should be related to the formation of fog or water droplets that adhere to the inner wall of chambers and related to its own lifespans (the lifespans of water vapor and aerosols are approximately 10 days, and that of ammonium sulfate is approximately 6 days).

On basis of the physical principle, the conditions required for the formation of fog are condensation nuclei, air cooling, and an increase in water vapor (humidity). If there are no condensation nuclei, the relative humidity must exceed 100%. In the atmospheric environment of autumn and winter, the formation of fog is mainly due to the increase in humidity and air particulate matters, the decrease in temperature. However, under our experiments, the increase in water vapor and $PM_{2.5}$ was the fog formation cause in the chambers of tap water and river water, but the fog formation cause in the chamber of ultrapure water was only the increase in water vapor because air temperature and pressure, the volume of air, and time and speed of humidification in three chambers are the same or similar.

In addition, when water vapor in air mass increases, the water vapor can make the air density decrease due to the lighter water vapor molecules, which may be the reason for expansion of these chambers at the end of experiments.

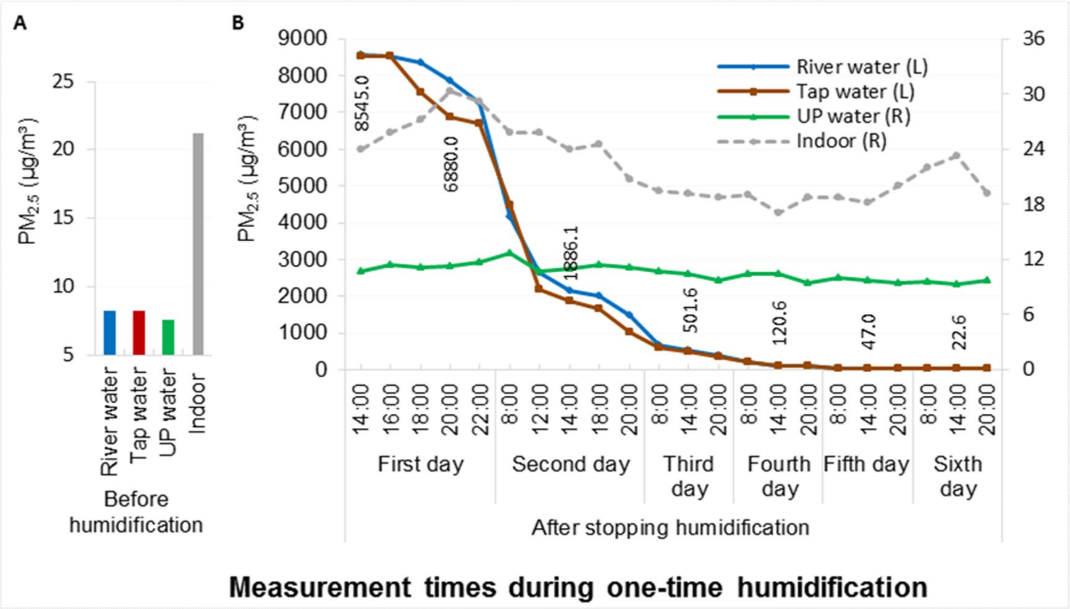

Figure 4. $PM_{2.5}$ concentration in three sealed chambers and in the surrounding room before humidification **(A)** and after stopping humidification **(B)** in one-time humidifying experiment.

For tap water, the $PM_{2.5}$ concentration averaged only 8.3 μg/m³ before humidification **(A)**. After stopping humidification **(B)**, the $PM_{2.5}$ concentration reached 8545.0 μg/m³ at 14:00 (i.e., 2 hours later) on the first day, but the $PM_{2.5}$ concentration gradually decreased from the second to sixth day (from 1886.1 to 22.6 μg/m³). In panel **B**, $PM_{2.5}$ concentrations between three types of water were different (DF=2, F=66.98, p <0.0001), and $PM_{2.5}$ concentrations between different measurement times (excluding ultrapure water) were also different (DF=21, F=83.44, p <0.0001). Further multiple comparisons (LSD) showed that $PM_{2.5}$ concentrations in tap water and river water were higher than those in ultrapure water (p <0.05), but there was no difference for $PM_{2.5}$ concentrations between tap water and river water (p >0.05); $PM_{2.5}$ concentrations on the first, second and third days were higher than the fourth, fifth and sixth days (p <0.05). The $PM_{2.5}$ concentration in

each sealed chamber was not affected by that in the surrounding room. $PM_{2.5}$ concentration was decreased to minimum after 6 days of stopping humidification. The marked numbers are tap water results.

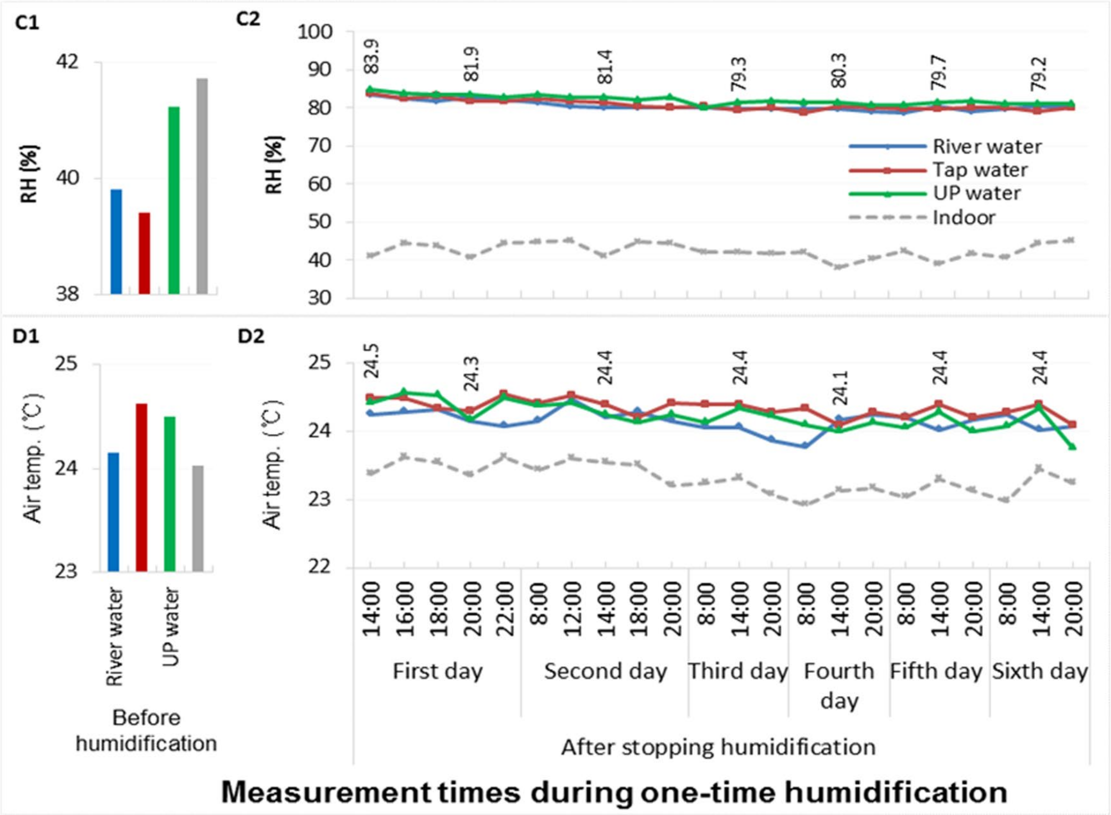

Figure 5. Changes in RH (**C1, C2**) and temperature (**D1, D2**) and physical phenomena in each sealed chamber in one-time humidifying experiment.

For tap water, the RH averaged 39.4% before humidification (**C1**). Both RH (**C2**) and temperature (**D2)** changed little after stopping humidification (from 83.9% to 79.2% and from 24.5 to 24.4°C, respectively). The humidity of ultrapure water is slightly high and the temperature of river water is slightly low. The difference in humidity and temperature between measurement times existed only between a few groups. The RH and temperatures in the sealed chambers were not affected by those in the room. The marked numbers are tap water results.

Physical phenomenon: Before humidification, each sealed chamber was dry, transparent, and lightly concave on side walls. The difference from the above five-day experiment was that the humidifying process resulted in slight fog at 60 minutes, dense fog at 120 minutes, and large fog droplets at 200 minutes (the droplet size was smaller for the ultrapure water fog). After stopping humidification, each sealed chamber experienced slight to moderate to large expansion and was humid and hot (with an unpleasant odor in the river water chamber).

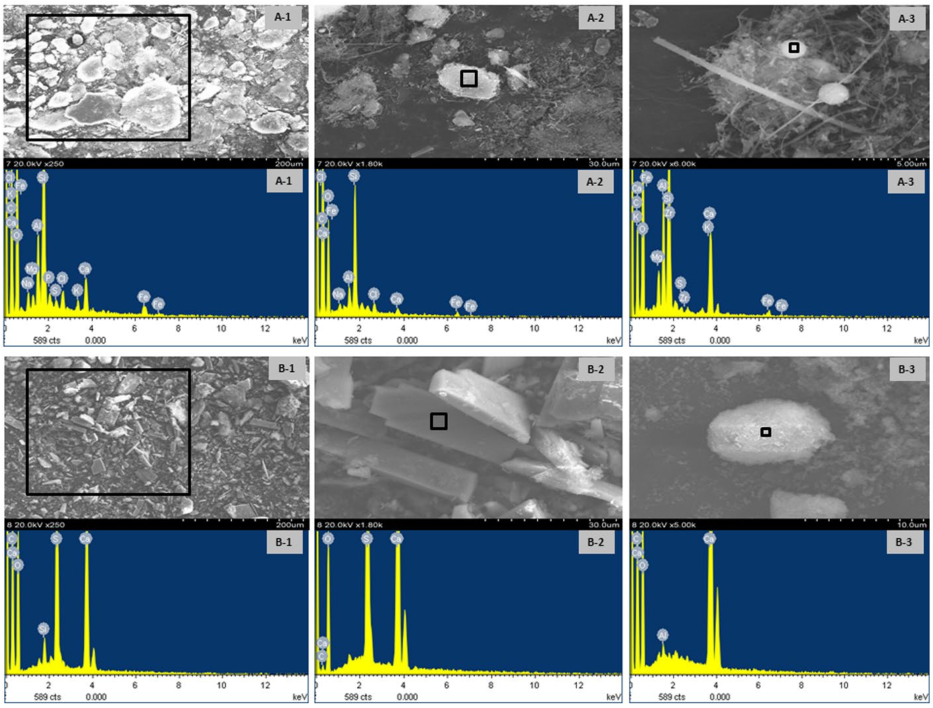

Figure 6. Residue composition analysis in the three types of water. River water **(A-1, A-2, A-3)** contained 16 elements, and the atomic percentages were O (65.87%), C (22.77%), Si (4.88%), Ca (0.71%), and Al, Fe, Mg, Na, Cl, K, S, F, P, Zr, Zn, Ti (0.03%); tap water **(B-1, B-2, B-3)** mainly contained eight elements, and the atomic percentages were O (66.79%), C (21.69%), Ca (5.05%), S (4.88%), and Si, Al, Cl, Zr (0.07%).

The atomic percentages for elements Ca, S and Si in the two types of water were different. Combining with the compound percentages and the results in Table 1, it could be inferred that the river water mainly contained carbonate and silicide, and the tap water mainly contained carbonate and sulphate.
